# Role of the *ABCA4* Gene Expression in the Clearance of Toxic Vitamin A Derivatives in Human Hair Follicle Stem Cells and Keratinocytes

**DOI:** 10.3390/ijms24098275

**Published:** 2023-05-05

**Authors:** Aneta Ścieżyńska, Krzysztof Łuszczyński, Marcin Radziszewski, Michał Komorowski, Marta Soszyńska, Natalia Krześniak, Kateryna Shevchenko, Anna Lutyńska, Jacek Malejczyk

**Affiliations:** 1 Department of Histology and Embryology, Medical University of Warsaw, 02-004 Warsaw, Poland; 2 Department of Medical Biology, National Institute of Cardiology, Stefan Cardinal Wyszyński State Research Institute, 04-628 Warsaw, Poland; 3 Department of Plastic and Reconstructive Surgery, Medical Centre of Postgraduate Education, Prof. W. Orlowski Memorial Hospital, 00-416 Warsaw, Poland

**Keywords:** *ABCA4* gene, keratinocytes, hair follicle stem cells, cell proliferation, cell differentiation, vitamin A derivatives, retinoids, all-trans-retinal

## Abstract

The *ABCA4* gene encodes an ATP-binding cassette transporter that is expressed specifically in the disc of photoreceptor outer segments. Mutations in the *ABCA4* gene are the main cause of retinal degenerations known as “*ABCA4*-retinopathies.” Recent research has revealed that *ABCA4* is expressed in other cells as well, such as hair follicles and keratinocytes, although no information on its significance has been evidenced so far. In this study, we investigated the role of the *ABCA4* gene in human keratinocytes and hair follicle stem cells for the first time. We have shown that silencing the *ABCA4* gene increases the deleterious effect of all-trans-retinal on human hair follicle stem cells.

## 1. Introduction

The *ABCA4* gene (OMIM 601691; GenBank: NG_009073.1), encoding an ATP-binding cassette transporter, which has long been thought to be a lipid flippase selectively expressed in the disc of outer segments of photoreceptors, that is, highly specialized and differentiated cells of the retina [1]. Mutations of the *ABCA4* gene are the cause of retinal degenerations called “*ABCA4*-retinopathies”, with the most prevalently inherited macular dystrophy called Stargardt disease (STGD1; MIM# 248200) [2]. Recent studies showed that *ABCA4* is also expressed in other cells apart from photoreceptors, such as retinal pigmented epithelium cells [3] or amniotic fluid-derived mesenchymal stromal cells [4]. Expression of the full-length *ABCA4* gene transcript and protein was also reported in hair follicles, and in keratinocytes [5].

Genes considered to be retina-specific are believed to perform an important role in skin homeostasis. It was noted that the human epidermis, dermis, and anagen hair follicles express a wide spectrum of retinal-specific genes, e.g., *opsin 2* (*OPN2, Rhodopsin*) and *opsin 3* (*OPN3, Panopsin, Encephalopsin*) [6,7,8]. Silencing of the *OPN3* in the outer root sheath cells of the hair follicle resulted in abrogated proliferation in these cells due to blue light treatment or led to a reduction in keratinocyte differentiation [9,10].

ABCA4 protein is responsible for the transport of retinoid intermediates of the visual cycle, mainly the all-trans-retinal (at-RAL) or its Schiff base conjugate with phosphatidylethanolamine, known as N-retinylidene-phosphatidylethanolamine (N-ret-PE), from the luminal side of the photoreceptor discs to the cytoplasm of the photoreceptors [1]. Once transported, at-RAL is reduced to vitamin A by trans-retinol dehydrogenases in the cytoplasm. Dysfunction of the ABCA4 transporter leads to the accumulation of N-ret-PE and all-trans-retinal in the disc membranes and its further accumulation into di-retinoid compounds, such as dihydropyridine N-retinylidene-N-retinyl-phosphatidylethanolamine (A2PE) and bis-retinoid N-retinyl-N-retinylidene ethanolamine (A2E). After that, they cannot be further metabolized and progressively accumulate as fluorescent lipofuscin deposits that exhibit phototoxic and cytotoxic properties [11].

The human epidermis contains two types of retinoids: retinol and retinyl esters and carotenoids (mostly β-carotene) [12]. Carotenoids, particularly β-carotene, the most abundant vitamin A precursor in humans, were thought to be solely processed into vitamin A in enterocytes or in the liver. The observation that bioconversion of [14C]-carotene to [14C]-retinol occurs also in cultured human melanocytes, and keratinocytes reignited the debate about whether topical carotenoids may act as cutaneous vitamin A precursors [13]. Microsomal enzymes catalyze the synthesis of retinol fatty acyl esters, the main form of vitamin A in the epidermis, that may be stored in keratinocytes [14]. This phase is catalyzed by two enzymes, acyl-CoA:retinol acyltransferase (ARAT) and lecithin:retinol acyltransferase (LRAT), whose expression increases in terminally differentiating keratinocytes [12,15]. In the epidermis, keratinocytes undergo spatiotemporal, highly controlled differentiation program, which is reflected by the presence of 5 epidermal layers called stratum basale (stratum germanitivum), stratum spinosum, stratum granulosum, stratum lucidium (only in thick skin), and stratum corneum [16]. Differentiating keratinocytes increase expression of stratum corneum markers, such as Involucrin (INV) and Loricrin, whereas stratum basale markers, such as keratin 5, decrease [16].

Interestingly, *ABCA4* and two other ABC-transporters, i.e., *ABCA1* and *ABCA12,* are highly and almost uniformly expressed throughout the hair follicle epithelium [17]; however, their role in proliferation, differentiation and migration of hair follicle epithelial cells is still poorly understood. To date, few experiments assessed the role of the *ABCA1* and *ABCA12* genes in the skin. It was indicated that mutations in the *ABCA1* gene are the cause of reduced cholesterol efflux from cells (Tangier disease) and may perform a role in the early senescence of human skin fibroblasts [18]. Other observations showed that mutations of the *ABCA12* gene are also involved in the skin pathology known as harlequin ichthyosis [19,20]. On the other hand, no information was provided for the third most expressed ABC transporter of hair follicle epithelium, *ABCA4*.

In this paper, we investigate the role of the *ABCA4* gene in human keratinocytes and hair follicle stem cells and show for the first time that *ABCA4* gene silencing enhances the toxic effect of at-RAL on human hair follicle stem cells.

## 2. Results

### 2.1. ABCA4 Protein Is Expressed in the Basal Layer of the Epidermis

**Figure 1:**
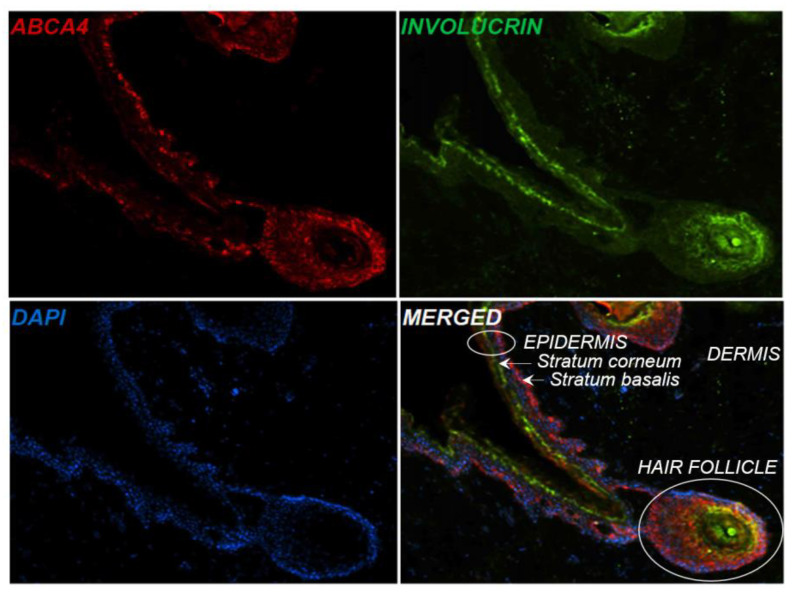
Localization of the ABCA4 protein in the epidermal layers. Immunofluorescence staining revealed expression of the ABCA4 protein (red) within the basal layer of the epidermis, compared to INVOLUCRIN (green), which is a marker of differentiating keratinocytes, expressed in the upper layers of the skin.

Immunofluorescence analysis of the ABCA4 protein localization within the normal human skin showed high expression of the ABCA4 protein in the epidermis, especially within the basal layer (Figure 1), contrary to the INVOLUCRIN, which is a marker of stratum corneum of the epidermis. ABCA4 protein was also highly expressed in human hair follicles, while low expression of the ABCA4 protein was noted in the dermis.

**Figure 2:**
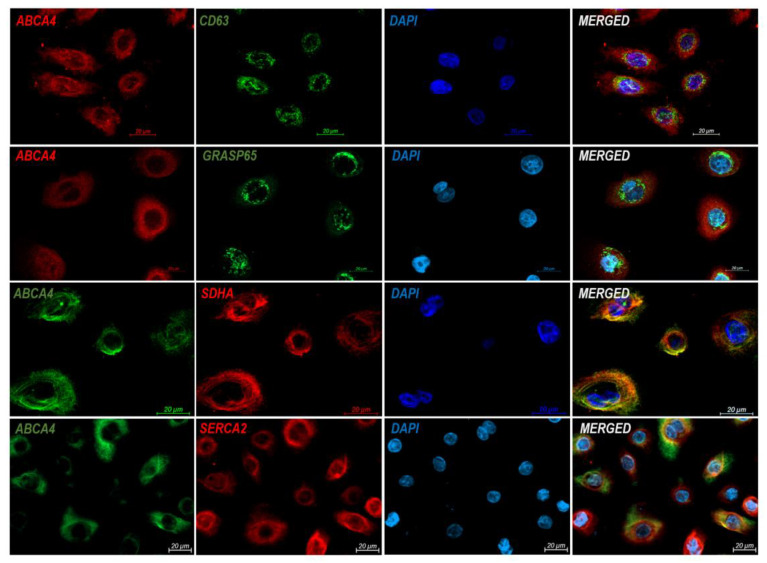
Expression of the ABCA4 protein in normal human keratinocytes. CD63, protein of late endosomal and lysosomal membranes; GRASP65, protein of Golgi apparatus; SDHA, protein of mitochondria; SERCA2, protein of the endoplasmic reticulum.

Keratinocyte immunostaining with several markers of cellular components, such as CD63 (*lysosome-associated membrane protein 3*, lamp3; component of the late endosomal and lysosomal membranes [21]), GRASP65 (*Golgi reassembly stacking protein*; peripheral protein of Golgi apparatus), SDHA (*succinate dehydrogenase complex flavoprotein subunit A*; complex of the mitochondrial respiratory chain), and SERCA2 (*sarco/endoplasmic reticulum Ca^2+^-ATPase*; protein of the endoplasmic reticulum) revealed variable cytoplasmic expression of the ABCA4 protein. Yellow/orange areas visible on merged pictures in Figure 2 indicate partial correspondence of the ABCA4 protein with the localization of the SERCA2 and SDHA proteins. In contrast, ABCA4 does not co-localize with the CD63 and GRASP65 proteins.

### 2.2. Treatment with All-Trans-Retinal induces Expression of the ABCA4 Gene in Human Hair Follicle Stem Cells

**Figure 3:**
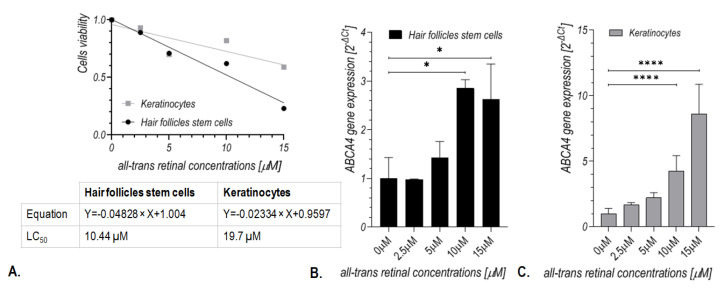
The effect of at-RAL concentrations (2.5, 5, 10, 15 μM) on cell viability (**A**) and *ABCA4* gene expression in human hair follicle stem cells (**B**) and keratinocytes (**C**). Human hair follicle stem cells: * *p*-value of 0.0196 (control vs. 10 µM) and 0.0465 (control vs. 15 µM). Keratinocytes: **** *p*-value < 0.0001.

As shown in Figure 3A, an increase in at-RAL concentrations decreased the viability rate of keratinocytes and hair follicle stem cells. Following a 24 h incubation, the half-maximal lethal concentration (LC50) for at-RAL was 10.44 μM and 19.7 µM for hair follicle stem cells and keratinocytes, respectively. *ABCA4* gene expression increased in accordance with at-RAL concentrations (2.5, 5, 10, and 15 µM), with a statistically significant difference beginning from 10 µM of at-RAL for both hair follicle stem (Figure 3B) cells and keratinocytes (Figure 3C).

**Figure 4:**
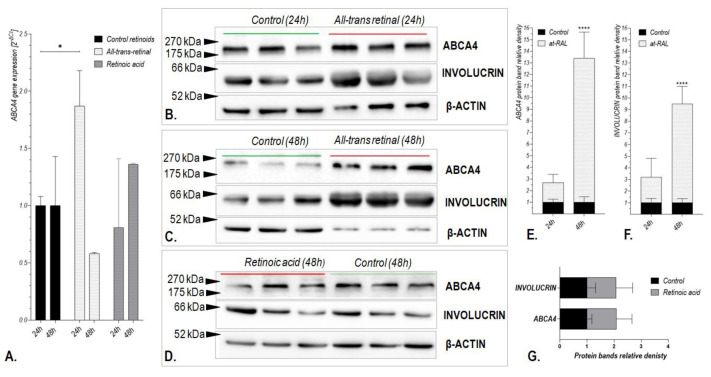
Expression of the *ABCA4* gene at mRNA and protein levels after treatment of hair follicle stem cells (HHF-SCs) with vitamin A derivatives: 10 μM all-trans-retinal (at-RAL) and 1 µM retinoic acid. *ABCA4* gene expression was assessed by qRT-PCR for HHF-SCs after 24 and 48 h of treatment with vitamin A derivatives (**A**). * *p*-value of 0.0157. Analysis of ABCA4 protein expression assessed by Western Blot for HHF-SCs after 24 and 48 h of treatment with 10 μM all-trans-retinal (**B**,**C**) and 1 µM retinoic acid (**D**). Relative densities of Western Blot bands for ABCA4 and Involucrin proteins after 24 and 48 h of treatment of HHF-SCs with 10 μM all-trans-retinal (**E**,**F**, respectively) and 1 µM retinoic acid (**G**). **** *p*-value < 0.0001.

Treatment of human hair follicle stem cells (HHF-SCs, n = 4) with selected vitamin A derivatives (10 μM all-trans-retinal and 1 µM retinoic acid) revealed that at-RAL induced *ABCA4* gene expression at both mRNA and protein levels (Figure 4). An increase in *ABCA4* mRNA expression was observed after 24 h from treatment with at-RAL (*p*-value of 0.0157; Figure 4A), while an increase in the ABCA4 protein level was sustained until 48 h after cell treatment with at-RAL (Figure 4B,C,E). Retinoic acid did not significantly induce *ABCA4* gene expression at either mRNA or protein level (Figure 4D,G).

**Figure 5:**
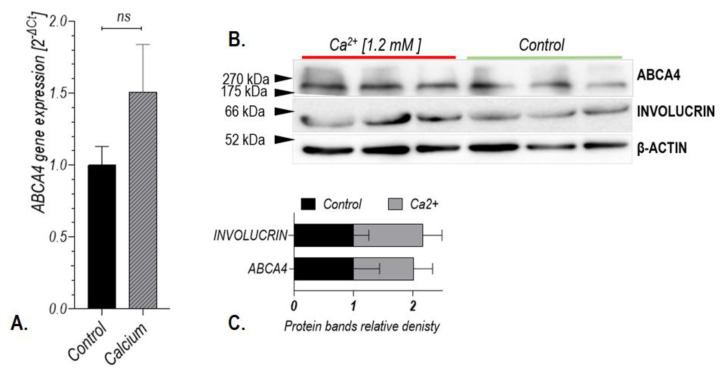
Expression of the *ABCA4* gene at mRNA (**A**) and protein (**B**) levels after 24 h of hair follicle stem cells treatment with calcium ions (1.2 μM). ns: not significant.

Increased expression of the ABCA4 protein after at-RAL (10 μM) treatment in human hair follicle stem cells positively correlated with the expression of involucrin (Figure 4B,C,F). Treatment of hair follicle stem cells with calcium ions (1.2 µM) for 24 h did not induce *ABCA4* gene expression in those cells (Figure 5).

### 2.3. ABCA4 Gene Silencing Enhances Toxic Effect of All-Trans-Retinal on Human Hair Follicle Stem Cells

**Figure 6:**
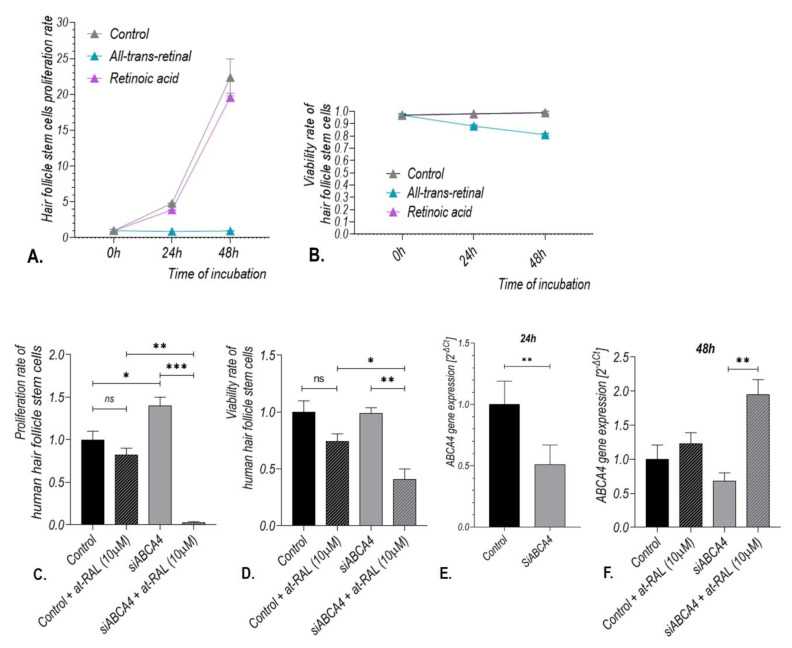
Effect of at-RAL treatment on proliferation, viability rate and *ABCA4* gene expression in human hair follicle stem cells. Cell proliferation rate (**A**) and viability (**B**) of human hair follicle stem cells after 24 h of incubation with at-RAL (10 μM) or retinoic acid (1 μM). Hair follicle stem cells proliferation rate (**C**) and viability (**D**) after 24 h of incubation with the *ABCA4* gene silencers (siABCA4) followed by 24 h treatment with at-RAL (10 μM). *ABCA4* gene expression in human hair follicle stem cells after 24 h of incubation with *ABCA4* gene silencers (siRNA) (**E**) followed by 24 h treatment with at-RAL (10 μM) (**F**). Proliferation rate: ns—not significant, * *p*-value of 0.0267, ** *p*-value of 0.0022, *** *p*-value of 0.0003; Viability rate: ns—not significant, * *p*-value of 0.0476, ** *p*-value of 0.0062; *ABCA4* gene expression: ** *p*-value of 0.0018 after 24 h of cell culture and ** *p*-value of 0.0076 after 48 h of cell culture.

Stimulation of HHF-SCs with at-RAL for 24 h significantly reduced the proliferation rate to 18% (*p*-value < 0.0001) and viability to 89%, even though the latter was not statistically significant (Figure 6A,B). Silencing of the *ABCA4* gene in human hair follicle stem cells increased cell proliferation rate compared to control (*p*-value of 0.0267) (Figure 6C) but had no influence on cell viability (Figure 6D). Interestingly, the proliferation rate of HHF-SCs incubated with *ABCA4* gene silencers for 24 h and subsequently treated with 10 μM at-RAL for 24 h was reduced to 0.03% compared with HHF-SCs treated with *ABCA4* gene silencers alone (*p*-value of 0.0003; Figure 6C). In those cells, viability was also significantly reduced (*p*-value of 0.0062; Figure 6D).

Reduction of the *ABCA4* gene expression after 24 h of incubation with *ABCA4* gene silencers was assessed by qRT-PCR (*p*-value = 0.0018; Figure 6E). Interestingly, treatment of HHF-SCs with 10 µM at-RAL for subsequent 24 h alleviated the reduction the *ABCA4* gene expression caused by *ABCA4* gene silencing (*p*-value of 0.0076; Figure 6F).

### 2.4. Treatment with All-Trans-Retinal Induced Expression of the ABCA4 Gene in Human Keratinocytes

**Figure 7:**
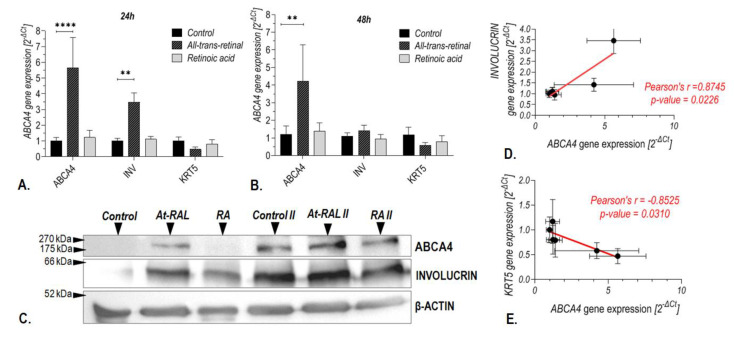
Expression of the *ABCA4* gene at mRNA and protein levels in keratinocytes treated with vitamin A derivatives. *ABCA4* gene expression after 24 (**A**) and 48 h (**B**) of treatment with 10 μM all-trans-retinal and 1 μM retinoic acid, assessed by qRT-PCR. Western Blot analysis of ABCA4 protein expression after 48 h treatment with 10 μM all-trans-retinal (at-RAL) and 1 μM retinoic acid (RA) (**C**). The plot of the correlation between *ABCA4* gene expression and *Involucrin (INV)* (**D**) or *KRT5* gene (**E**). *ABCA4* gene expression: **** *p*-value < 0.0001 after 24 h of cell culture and ** *p*-value of 0.005 after 48 h of cell culture; INV expression: ** *p*-value of 0.0014 after 24 h of cell culture.

A pronounced induction of *ABCA4* mRNA expression was observed in keratinocytes after 24 (Figure 7A) and 48 h (Figure 7B) of treatment with 10 μM at-RAL (*p*-value < 0.0001 and *p*-value = 0.005, respectively). An increase was also detected at the protein level after 48 h of treatment with at-RAL (Figure 7C). In contrast, treatment with 1 μM retinoic acid (RA) did not significantly induce *ABCA4* gene expression at mRNA (Figure 7A,B) or protein level (Figure 7C). An increased expression of ABCA4 protein after 10 μM at-RAL treatment positively correlated with *INV* expression in human keratinocytes (Figure 7D) and negatively with expression of the *KRT5* gene (Figure 7E).

**Figure 8:**
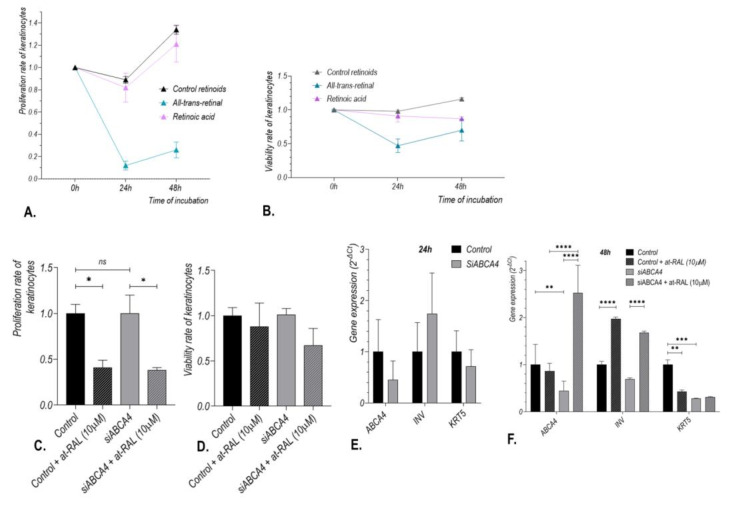
Effect of treatment with vitamin A derivatives on proliferation, viability rate, and *ABCA4* gene expression in normal human keratinocytes. Cell proliferation rate (**A**) and viability (**B**) of keratinocytes after 24 h of incubation with at-RAL (10 μM) or retinoic acid (1 μM). Proliferation rate (**C**) and viability (**D**) of keratinocytes after 24 h of incubation with the *ABCA4* gene silencers (siABCA4) followed by 24 h treatment with at-RAL (10 μM). *ABCA4* gene expression in keratinocytes after 24 h of incubation with *ABCA4* gene silencers (siRNA) (**E**) followed by 24h treatment with at-RAL (10 μM) (**F**). Proliferation rate: ns – not significant, * *p*-value of 0.0265 and 0.0223, respectively; *ABCA4* gene expression: ** *p*-value of 0.0072 and **** *p*-value < 0.0001 after 48 h of cell culture; *INV* gene expression: **** *p*-value < 0.0001 after 48 h of cell culture; *KRT5* gene expression: ** *p*-value of 0.0061 and *** *p*-value of 0.0007 after 48 h of cell culture.

Silencing of the *ABCA4* gene in normal human keratinocytes did not influence cell proliferation rate or viability compared to control (Figure 8C,D). The proliferation rate of keratinocytes incubated with *ABCA4* gene silencers for 24 h and subsequently treated with 10 μM at-RAL for 24 h was reduced to about 45% compared to keratinocytes treated with *ABCA4* gene silencers alone (*p*-value of 0.0223; Figure 8C); however, a significant reduction in cell proliferation after at-RAL incubation was also observed in cells not pre-treated with *ABCA4* gene silencers (*p*-value of 0.0265; Figure 8C). In cells treated with 10 μM at-RAL, cell viability was also reduced, even though this difference does not reach scientific significance (Figure 8D).

Reduction of *ABCA4* gene expression in normal human keratinocytes after 24 h of incubation with *ABCA4* gene silencers was assessed by qRT-PCR (Figure 8E). Interestingly, treatment of keratinocytes with 10 µM at-RAL for subsequent 24 h alleviated the reduction of *ABCA4* gene expression caused by *ABCA4* gene silencing (*p*-value < 0.0001; Figure 8F).

## 3. Discussion

Following the identification of full-length transcripts of the *ABCA4* gene in human skin cells and hair follicles [5], the role of the *ABCA4* gene in human skin homeostasis needs to be further elucidated. In this paper, we have reported for the first time the behavior of hair follicle stem cells and keratinocytes in response to treatment with at-RAL and *ABCA4* gene silencing.

First, we verified whether cell treatment with at-RAL, a well-known substrate of the *ABCA4* gene in photoreceptor cells, influenced *ABCA4* gene expression in human skin cells. Indeed, we discovered that treatment with 10 µM at-RAL increases *ABCA4* mRNA and protein expression in the human hair follicle stem cells and keratinocytes (Figure 4 and Figure 7). Since, in human cells, at-RAL can be reversibly reduced into retinol by dehydrogenases or irreversibly oxidized into all-trans-retinoic acid [22], we investigated whether stimulation of *ABCA4* expression is aldehyde-specific. We found no substantial activation of the *ABCA4* gene in hair follicle stem cells (Figure 4) or keratinocytes (Figure 7) after 24 h of incubation with 1 µM retinoic acid (concentration frequently used during keratinocyte cell culture), which indicates that the observed changes were specific to at-RAL. After at-RAL treatment, expression of the *ABCA4* gene increased nearly twofold (Figure 4) in HHF-SCs, while in keratinocytes, the level of *ABCA4* mRNA expression increased nearly sixfold (Figure 7). Immunolocalization staining showed that the *ABCA4* gene is strongly expressed in the basal layer of the epidermis and in hair follicle cells, suggesting that the *ABCA4* may be involved in cell proliferation and stem cell activity. The self-renewal potential of the epidermis is sustained by the existence of stem cell populations located in the basal layer of the epidermis or the hair follicular stem cells located in the hair bulges [23]. Descendants of stem cells, the so-called transit-amplifying (TA) cells that withdraw from the cell cycle after a few rounds of division undergo a terminal differentiation program to form the dead, cornified cells (corneocytes) of lipid-enriched stratum corneum [24,25]. High expression of the *ABCA4* gene in hair follicles suggests that its endogenous substrate perform an important role in hair follicle function. It is possible that *ABCA4* shields hair follicle stem cells from the accumulation of metabolic intermediates, maintaining their progeny features, as it was observed in the case of another member of the ABC protein family, the ABCG2 transporter [26]. Indeed, based on obtained results, we observed that the natural substrate of the *ABCA4* gene significantly reduces proliferation rates in HHF-SCs, and silencing of the *ABCA4* gene increases the toxicity of at-RAL (Figure 6) on proliferation and cell viability. Interestingly, *ABCA4* gene silencing without at-RAL treatment increases the proliferation rate of HHF-SCs, while in keratinocytes proliferation rate after *ABCA4* gene silencing remains unchanged (Figure 6 and Figure 8). This aspect will need to be further investigated; however, such differences might be linked to the difference in the proliferation rates of HHF-SCs as compared to keratinocytes. While HHF-SCs have a population doubling time of approximately 24–26 h, keratinocytes proliferate at a rate of up to 60 h [27] and readily commence the differentiation process, which results in cell death. Moreover, it might also suggest that the *ABCA4* gene has distinct actions in cells at various levels of differentiation. Indeed, Huang et al. observed that repression of the *ABCA4* gene of self-renewable, multipotent stem cells, that is, amniotic fluid-derived mesenchymal stromal cells, increased their proliferation rate [4,28]. On the other hand, *ABCA4* knock-down of retinal pigment epithelium (RPE) cells, terminally differentiated cells with a doubling time of 55–65 h, significantly reduced their proliferation rate, induced apoptosis, and inhibited migration [29]. Highly proliferative cells are more prone to be affected by cytotoxic agents than fully differentiated cells. This agrees with the estimated value of LC50 for human hair follicle stem cells, which is lower than the value obtained for keratinocytes.

Moreover, we observed that at-RAL treatment greatly increased the expression of the *ABCA4* gene in keratinocytes and that following at-RAL treatment, the expression of the *ABCA4* gene in keratinocytes positively correlated with the expression of the *INV* gene (marker of differentiation) (Figure 7) and negatively correlated with the *KRT5* gene activity, which is a marker of undifferentiated basal stem cells and reflects their proliferative capacity [30]. These results were sustained in concordance with the observation that retinal was transformed into retinoic acid more slowly in non-differentiating keratinocytes than in differentiating ones [31], and only keratinocytes that already committed to terminal differentiation can express an enzymatic system catalyzing the conversion of retinol into retinoic acid. Therefore, differentiating keratinocytes were found more efficient at neutralizing at-RAL than non-differentiating keratinocytes [31]. Moreover, at-RAL treatment in keratinocytes counteracts *ABCA4* gene silencing, and the increase in the *ABCA4* gene expression may be sufficient to protect cells against at-RAL cytotoxicity.

The human epidermis contains endogenous retinoids (retinol and retinyl esters) and carotenoids (mainly β-carotene) [12]. Keratinocytes and melanocytes can convert beta-carotene into retinol (vitamin A) [13]. Too little and too much retinol may be harmful to skin cells. Retinoic acid affects hair follicle stem cells in a dose-dependent manner and is involved in the regulation of the hair follicle cycle [32]. Additionally, retinoic acid synthesized in keratinocytes enhances melanocyte stem cells’ development into melanocytes. Otherwise, melanosomes are transported from melanocytes to keratinocytes in a retinal-dependent way [32]. Retinol is oxidized into retinal and subsequently into retinoic acid [32]. The metabolism of vitamin A derivatives is confined to different cellular compartments. While reduction in retinal to retinol may occur in the cytosol, regulated by several aldo–keto reductases, or in endoplasmic reticulum membranes, regulated by microsomal retinaldehyde reductases (RDHs), retinaldehyde oxidation to retinoic acid occurs in the cytosol [33]. Interestingly, immunostaining of normal human keratinocytes revealed expression of the ABCA4 protein in an area partially corresponding to the location of endoplasmic reticulum and mitochondria (Figure 2). ABCA4 protein may be localized within mitochondria-associated endoplasmic reticulum membranes (MAMs), which promote the local synthesis of phosphatydylocholine, phosphatidylethanolamine (PE), and phosphatidylcholine [34], but this subject must be investigated further. PE, the physiological lipid substrate of the ABCA4 transporter, is the second most common phospholipid in mammalian cells and is located predominantly in the inner leaflet of cellular membranes, notably prevalent in the inner mitochondrial membrane [35].

In photoreceptors, in order to prevent the buildup of toxic di-retinoid compounds, at-RAL (in conjugation with PE) is translocated by the ABCA4 transporter from the lumen of the photoreceptor discs to the cytoplasm of photoreceptors. In keratinocytes, the ABCA4 transporter may also translocate at-RAL from the lumen of the endoplasmic reticulum to the cytoplasmic side. Insufficient clearance of retinal normally occurring in keratinocyte culture, following the *ABCA4* gene silencing, may lead to accumulation of at-RAL, which induces mitochondria-associated reactive oxygen species (ROS) production and has the potential to cause endoplasmic reticulum (ER) stress [36]. Physiological levels of ER stress activate the unfolded protein response (UPR), which is essential for maintaining proper functions of ER and induction of the keratinocyte differentiation pathway (which may explain increase in the expression of involucrin after treatment with at-RAL). In response to excessive or prolonged ER stress, however, the UPR can activate a programmed cell death (apoptosis) pathway [37]. Endoplasmic reticulum stress may also affect the export of PE from mitochondria to ER [38].

Interestingly, Wiley et al. showed a truncated version of the ABCA4 protein (about 70 kDa) in human keratinocytes. This band was also present in the human retina, which served as a control. In that paper, ABCA4 protein of molecular weight of c.a. 250 kDa was also present, albeit faintly [39]. We also found (however, with use of different antibodies) smaller bands of the ABCA4 protein, in addition to the major c.a.250 kDa band, even though they were not present in all samples (Supplementary Figure S1). Further research is needed to determine the source of this occurrence, whether extra bands are generated by the existence of splice variants or during protein cleavage or degradation, and whether this phenomenon is related to keratinocytes differentiation and aging. Furthermore, further protein-level analysis of the samples that underwent *ABCA4* gene silencing will be required. It should be highlighted, however, that labeling the endogenous expression of the ABCA4 protein in keratinocytes may be difficult due to its relatively low expression level and/or antibody-binding properties. Nonetheless, data acquired from retinal pigmented epithelium (RPE) cells shows that, despite the fact that the total ABCA4 protein of the RPE is only around 1% of total ABCA4 in the neural retina, ABCA4 function in RPE is not less important when compared to the retina [3]. Additionally, as some ABCA4 mutants have been found to be retained within the endoplasmic reticulum of photoreceptors or HEK 293T cells [40,41], functional activity of the ABCA4 transporter in human skin cells must be confirmed.

## 4. Materials and Methods

### 4.1. Tissue Collection and Cell Cultures

Normal human keratinocytes were isolated from skin samples obtained from five healthy individuals subjected to aesthetic breast reduction or aesthetic abdominoplasty in the Department of Plastic Surgery, Medical Centre of Postgraduate Education, Orlowski Memorial Hospital in Warsaw. The study was approved by the Ethics Committee at the Medical Centre of Postgraduate Education, Orlowski Memorial Hospital in Warsaw (87/PB/2020 obtained 15 June 2020). Keratinocytes were isolated from skin explants according to the method described in detail by Guo et al. [42]. Briefly, skin explants were cut into 1 mm fragments and inserted into 6-well plates. Skin explants that adhered to the plate surface were covered with Dulbecco’s Modified Eagle Medium (DMEM) supplemented with 20% Fetal Bovine Serum (FBS) (both from ThermoFisher Scientific Inc., Waltham, MA, USA) and cultured under standard cell culture conditions at 37 °C in 5% CO_2_ in air atmosphere. After approximately two days, primary keratinocytes growing from skin explants were collected after incubation in TrypLe solution (ThermoFisher Scientific Inc., Waltham, MA, USA) for 10 min in 37 °C and subsequently cultured in keratinocyte culture medium (KGM2 media, PromoCell, Heidelberg, Germany) under standard cell culture conditions. For subsequent experiments, cells at the third passage were routinely used. Human hair follicle stem cells (HHF-SCs) were purchased from Celprogen (Huissen, Netherlands) and cultured in Human Hair Follicle Stem Cell Complete Media (HHF-SCs CM) with Serum (Celprogen). Experiments performed for HHF-SCs were carried out in four replicates (n = 4).

### 4.2. ABCA4 Gene Silencing

**Figure 9:**
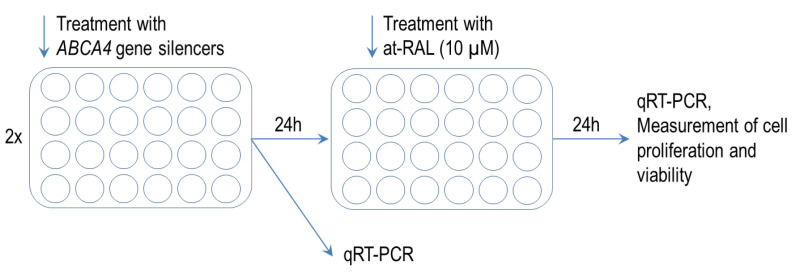
Experimental timeline of the *ABCA4* gene silencing.

Normal keratinocytes and human hair follicle stem cells were seeded in a 24-well plate at a density of 3.5 × 10^4^ cells/well and 1.25 × 10^4^ cells/well and cultured in KGM2 or HHF-SCs CM, respectively. After 24 h, culture media were replaced with appropriate culture media supplemented with Lipofectamine suspended in OptiMEM (ThermoFisher Scientific Inc.) and 12.5 μM *ABCA4* gene silencer no. 4,427,037 (ID: s536444) and 12.5 μM *ABCA4* gene silencer no. 4,392,422 (ID: s863, both from ThermoFisher Scientific Inc.) or with 25 μM Silencer Select Negative Control siRNA no. 4390843; (ThermoFisher Scientific Inc.), which served as a negative control. The success rates of transfections with *ABCA4* gene siRNAs were evaluated by quantitative reverse transcription PCR (qRT-PCR). For selected experiments, 24 h after *ABCA4* gene silencing, cells were stimulated with 10 μM at-RAL (Figure 9).

### 4.3. Proliferation and Viability Assays

Normal human keratinocytes and hair follicle stem cells were seeded in a 24-well plate at a density of 3.5 × 10^4^ cells/well and 1.25 × 10^4^ cells/well and cultured in KGM2 or HHF-SCs CM, respectively. Subsequently, the cells were cultured in fresh media supplemented with 10 μM all-trans-retinal (at-RAL; Sigma-Aldrich, St. Louis, MO, USA), 1 μM retinoic acid (Sigma-Aldrich) or 1.2 mM CaCl_2_ (PromoCell). During two following days of cell culture, the HHF-SCs and keratinocytes were detached by incubation in TrypLe solution for 5 min at 37 °C and their number and viability were analyzed with the use of the image based-immunofluorescence cell counter ADAM-MC (NanoEntek, Seoul, Republic of Korea).

### 4.4. Isolation of Total RNA and cDNA Synthesis

Total RNA was isolated with Total RNA Mini Plus Concentrator (A&A Biotechnology, Gdansk, Poland) according to the manufacturer’s guidelines. Normalized concentrations of RNA were reversely transcribed into the cDNA with the High Capacity cDNA Reverse Transcription Kit (ThermoFisher Scientific Inc.) with oligo dT primers according to the manufacturer’s instruction.

### 4.5. Quantitative Reverse Transcription PCR (qRT-PCR)

*ABCA4* gene expression was evaluated by qRT-PCR on an ABI 7500FAST theromocycler (Applied Biosystems, Foster City, CA, USA) with the SensiFASTTM Probe Lo-ROX Kit (Bioline, Luckenwalde, Germany) and specific TaqMan Probes for the *ABCA4* gene (Hs00979586_M1; ThermoFisher Scientific Inc.), *INVOLUCRIN (INV)* gene (Hs00846307_S1; ThermoFisher Scientific Inc.), and *KRT5* gene (Hs00361185_M1; ThermoFisher Scientific Inc.), according to the manufacturer’s instructions. As an internal control, *TBP* gene expression (TATA-box binding protein, Hs00427620 m1) was measured. A relative level of mRNA expression was calculated with Applied Biosystems™ Analysis Software, Relative Quantification Analysis Module, VERSION 4.3 (ThermoFisher Scientific Inc.) by the ΔCt method.

### 4.6. Western-Blot Analysis

Lysates of cultured HHF-SCs and keratinocytes were prepared by homogenization in a buffer containing 150 mM NaCl, 1% Triton X-100, 0.5% sodium deoxycholate, 0.1% SDS, and 50 mM Tris (pH 8.0). Protein concentration was determined using Pierce BCA Protein Assay Kit (ThermoFisher Scientific Inc.). Subsequently, 25 µg of proteins were separated by SDS–8% PAGE, transferred onto a nitrocellulose membrane (Amersham Protran 0.45 NC), blocked with 5% milk solution in TBST, and subsequently incubated at 4 °C overnight with 1:250 rabbit anti-ABCA4 (cat. no. PA5-87983, Thermo Fisher Scientific Inc., as described previously [5]), 1:750 mouse anti-Involucrin (cat.no MA5-11803, Thermo Fisher Scientific Inc.), or 1:5000 mouse anti-β-actin (cat. no. A2228, Sigma-Aldrich, St. Louis, MO, USA). Secondary HRP-conjugated goat anti-rabbit IgG antibodies (cat. no. GTX213110-01, GeneTex, CA, USA) or rabbit anti-mouse IgG antibodies (cat. no. GTX213111-01, GeneTex) were used. The signal was detected with SuperSignal West Pico PLUS Chemiluminescent Substrate (Thermo Fisher Scientific Inc.) and visualized with the use of the Syngene GBox Chemi XX9 System (Syngene, Cambridge, UK).

### 4.7. Immunofluorescence Staining

For immunofluorescent staining, skin fragments were snap frozen in OCT Leica Tissue Freezing Medium (cat. no. #14020108926, Wetzlar, Germany) and cut into 10 µm sections in Leica CM 1860 cryostat (Wetzlar, Germany), whereas the in vitro growing keratinocytes were seeded onto cell culture slides (cat no. #07-2101, Biologix, Shandong, China) and cultured in the KGM2 medium for 24 h under standard conditions. Skin fragment cryosections and cultured cells were fixed in 4% paraformaldehyde for 20 min and permeabilized in 0.1% Triton X-100 (ThermoFisher Scientific Inc.) for 3 min at room temperature. The specimens were washed three times in Phosphate buffered saline (PBS, ThermoFisher Scientific Inc.) and incubated in the blocking solution (3% BSA in PBS) for 30 min at room temperature. Then, the specimens were incubated for 1 h at room temperature in mouse anti-Involucrin (cat. no. MA5-11803, ThermoFisher Scientific Inc.), rabbit anti-ABCA4 (cat. no. PA5-87983, ThermoFisher Scientific Inc.), or mouse anti-ABCA4 monoclonal antibody (cat.no. GTX82741, GeneTex), and rabbit anti-SERCA2 polyclonal antibody (cat. no. GTX55790, GeneTex), mouse anti-GRASP65 monoclonal antibody (cat. no. MA5-25148, ThermoFisher Scientific Inc.), rabbit anti-SDHA monoclonal antibody (cat. no. ab240098, Abcam, Cambridge, UK), or mouse anti-CD63 monoclonal antibody (cat. no. sc-365604, Santa Cruz Biotechnology Inc., Dallas, TX, USA). For detection of the primary antibodies, Alexa Fluor 594 goat anti-rabbit, and Alexa Fluor 488 goat anti-mouse (all from ThermoFisher Scientific Inc.) secondary antibodies were used. Dilutions of 1:100 or 1:200 were used for primary and secondary antibodies, respectively. Cells were visualised using LSM 710 NLO confocal microscope (Zeiss, Jena, Germany) in the Laboratory of Confocal Microscopy, Chair and Department of Experimental and Clinical Physiology, Medical University of Warsaw.

### 4.8. Statistical Analysis

GraphPad Prism 5 (GraphPad Software, La Jolla, CA, USA) was used for data processing. Statistical significance was tested with the non-parametric two-way analysis of variance (ANOVA), with Tukey’s test for multiple comparisons where available. Values of *p* < 0.05 were considered significant. For the measurement of Western blot bands densities, Image J software (U. S. National Institutes of Health, Bethesda, MD, USA; imagej.nih.gov, accessed on 22 April 2023) has been used [43].

## 5. Conclusions

We showed for the first time that at-RAL treatment increases *ABCA4* gene expression in keratinocytes and hair follicle stem cells. We found that an increase in the *ABCA4* gene expression in keratinocytes correlates with an increase in *INV* gene expression, and reduced expression of the *KRT5* gene. Additionally, *ABCA4* gene silencing increases the at-RAL treatment’s toxic effects on human hair follicle stem cells.

## Data Availability

Specific data are available from the authors upon request.

## Supplementary Materials

The following supporting information can be downloaded at: https://www.mdpi.com/article/10.3390/ijms24098275/s1.
