# Supplementary material for: Role of the ABCA4 Gene Expression in the Clearance of Toxic Vitamin A Derivatives in Human Hair Follicle Stem Cells and Keratinocytes
Source: Int J Mol Sci. 2023 May 5;24(9):8275. doi: 10.3390/ijms24098275 (PMC10179012; doi:10.3390/ijms24098275)
Supplement: Supplementary file 1 [file ijms-24-08275-s001.zip › ijms-2338933-supplementary.pdf]

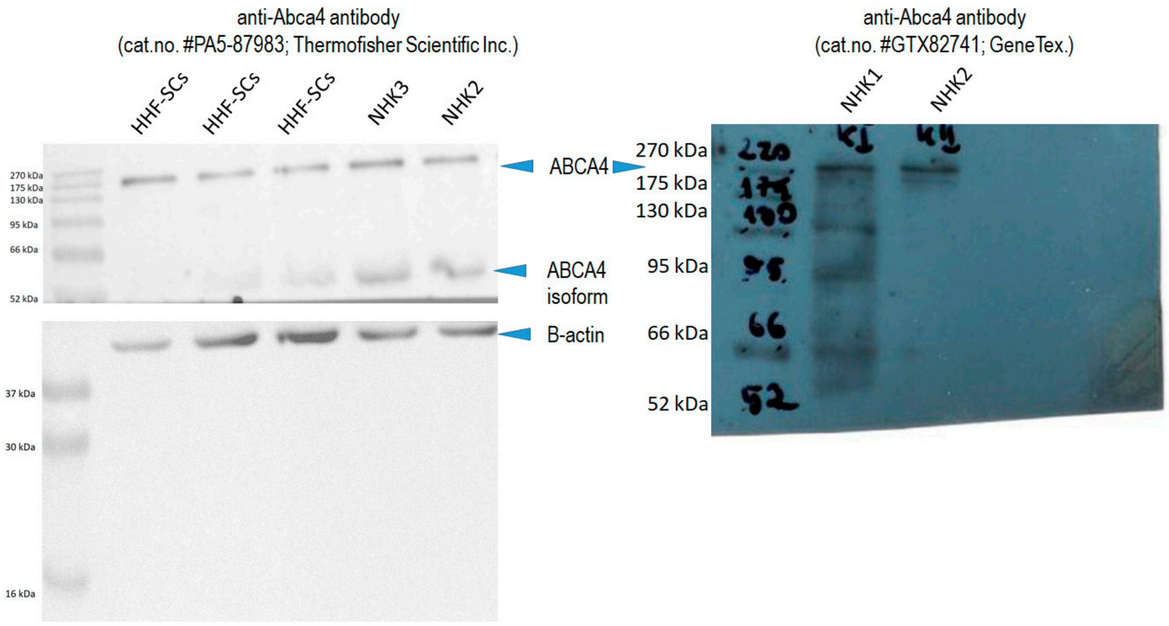

Supplementary Figure S1: Antibody specificity validation. Western Blot images revealed presence of the main antibody band of aprox. 256 kDa. In some samples additional bands of smaller molecular weight may be observed, both in human hair follicle stem cells (HHF-SCs), and keratinocytes obtained from different patients (NHK). NHK2, that was tested in Western blot presented in previous manuscript (Scieżyńska et al. Molecular Analysis of the ABCA4 Gene Mutations in Patients with Stargardt Disease Using Human Hair Follicles. Int J Mol Sci.) serves as a positive control.
